# A qualitative study of the interactions among the psychosocial work environment and family, community and services for workers with low mental health

**DOI:** 10.1186/1471-2458-13-796

**Published:** 2013-09-03

**Authors:** Catherine R Mackenzie, Dominic Keuskamp, Anna M Ziersch, Fran E Baum, Jennie Popay

**Affiliations:** 1 Southgate Institute for Health, Society & Equity, Flinders University, Adelaide, Australia; 2 Division of Health Research, Lancaster University, Lancaster, UK

**Keywords:** Psychosocial work environment, Life domains, Work-family, Socio-ecological model, Mental health

## Abstract

**Background:**

The psychosocial work environment can benefit and harm mental health. Poor psychosocial work environments and high level work-family conflict are both associated with poor mental health, yet little is known about how people with poor mental health manage the interactions among multiple life domains. This study explores the interfaces among paid work, family, community and support services and their combined effects on mental health.

**Methods:**

We conducted 21 in-depth semi-structured interviews with people identified as having poor mental health to examine their experiences of paid employment and mental health and wellbeing in the context of their daily lives.

**Results:**

The employment-related psychosocial work environment, particularly workplace relationships, employment security and degree of control over hours, strongly affected participants’ mental health. The interfaces among the life domains of family, community and access to support services suggest that effects on mental health differ according to: time spent in each domain, the social, psychological and physical spaces where domain activities take place, life stage and the power available to participants in their multiple domains. This paper is based on a framework analysis of all the interviews, and vignettes of four cases. Cases were selected to represent different types of relationships among the domains and how interactions among them either mitigated and/or exacerbated mental health effects of psychosocial work environments.

**Conclusions:**

Examining domain interactions provides greater explanatory capacity for understanding how people with low mental health manage their lives than restricting the research to the separate impacts of the psychosocial work environment or work-family conflict. The extent to which people can change the conditions under which they engage in paid work and participate in family and social life is significantly affected by the extent to which their employment position affords them latitude. Policies that provide psychosocial protections to workers that enable them to make changes or complaints without detrimental repercussions (such as vilification or job loss) and increase access to welfare benefits and support services could improve mental health among people with paid work. These policies would have particularly important effects for those in lower socioeconomic status positions.

## Background

### Introduction

Participation in paid work can benefit and harm mental health [1,2]. Numerous studies have examined a range of mental health effects produced by the psychosocial work environment and the conflict and/or facilitation between paid work and family life [3-5]. While some ‘work-life balance’ research has acknowledged interactions among paid work, family, civic and social life [6-8] there has been little research on the impact of specific domains outside paid work on workers’ mental health and wellbeing, while also taking the work environment into account [9]. This paper reports on the findings of in-depth interviews that explored workers’ experiences of the psychosocial work environment in relation to mental health, and the ways in which various other life domains interact to support or undermine their mental health.

### The relationship between the psychosocial work environment and mental health

Much research has investigated associations between the psychosocial environment experienced by workers and mental health [10-12]. The most common theoretical frameworks that researchers have employed to examine ways in which paid work affects mental health are Karasek’s demand-control model [13,14] and Siegrist’s effort-reward imbalance model [15]. These approaches have been used to explore workplace stressor-strain relationships to understand which and to what extent psychosocial stressors associated with paid work lead to physical or mental strain. The psychosocial work environment is defined by Bambra [10 p.74] as:

A collective way of referring to the psychological and social influences on health such as time pressure, monotonous work, social reciprocity, job control and autonomy, fairness, work demands, job security, as well as social contact between co-workers and supervisors.

A range of components of the psychosocial work environment have been linked to mental health outcomes including wages and conditions, job control and autonomy, work demands and job security [2,16,17]. Consistent with the international literature, Australian research indicates that the quality of the psychosocial environment people experience in paid work is strongly associated with mental health [18-20]. Studies have shown that people perceive stress as an important cause of mental ill-health [21,22] and that people are more likely to consider paid work to be the main source of the stress they experience when they are asked to attribute their stress to various life domains [12,22]. While stressors vary in their impact across occupations, interpersonal conflict has been shown to be the most pervasive [12].

The mental health impacts of paid work are unevenly distributed across the social gradient [23,24]. People in lower status occupations experience higher strain and, because they have less control over their working conditions, are less able to instigate changes that may reduce workplace stress than those in higher status occupations [10]. Similarly, people working in poor psychosocial environments that are commonly associated with low status occupations (e.g. low-skilled, low-paid jobs) experience poorer mental health than those in better quality jobs [18,19,25].

### Paid work and other life domains

Research suggests that promoting ‘work-life balance’ is important to protecting mental health and wellbeing for people in paid employment and by corollary improving productivity for organisations [6,26]. A recent review defined work-life balance as: ‘the individual perception that work and non-work activities are compatible and promote growth in accordance with an individual’s current life priorities’ [6 p.326]. Inherent in this definition is the dichotomy set up between work and ‘non-work' (i.e. paid work and everything else) - a dichotomy that fails to acknowledge that much activity outside the labour market is appropriately understood as unpaid work. While some scholars have used border [27] or boundary [28] theories to overcome the arbitrary separation between paid work and unpaid work by emphasising permeability and malleability between life domains, those domains are still typically conceptualised as home (or family) and work, albeit within their socio-political contexts [28].

In one attempt to break away from dualistic approaches to understanding how people manage multiple domains, Voydanoff [8] uses Brofenbenner’s ecological model of human development [29] to understand domain interactions within and among paid work, family and community. Voydanoff argues that people have a range of demands within each domain (e.g. work load in the work domain, caring responsibilities in the family domain) and resources (e.g. social networks in the community domain, spousal support in the family domain). Pocock and colleagues [30] add *time*, *space*, *life-stage* and *power* to Voydanoff’s model, expanding the ways in which life domains interact to influence mental health. We draw on Voydanoff’s model and Pocock and colleagues’ extension of it, to examine the interactions between these domains in the lives of workers with low mental health.

### Managing mental health impacts

Studies addressing occupational stressors and strains have mainly been directed at building individuals’ coping strategies, rather than at organisational change, in spite of evidence that management strategies and organisational culture are important for the mental health of workers [31,32]. Research that has examined coping strategies indicates that ‘talking to others, taking action to prevent stressors from occurring, and withdrawing into non-work activities’ are common [12 p.103]. Fossey and Harvey [33] found in a meta-synthesis of workforce experiences of people using mental health services that the types of workplace and mental health service structures and supports that are helpful for people with mental illness or disability include: promoting positive work environments, providing supports across settings (workplace, family/carers and mental health services) and removing systemic barriers such as policies that reduce income support upon returning to paid employment.

### This study

Research has rarely investigated the impact of specific domains outside paid work (e.g. home, community) on workers’ mental health and wellbeing, while also taking the psychosocial work environment into account [9]. The current study addresses the following questions: 1) how is the psychosocial environment associated with paid work perceived to affect mental health? 2) how do life domain interactions support or undermine mental health? and 3) what strategies do people use to protect their mental health? To answer these questions, we used data from 21 in-depth semi-structured interviews with people with low mental health to explore their experiences and perceptions of participation in the paid workforce in relation to their mental health in the context of their everyday lives.

## Methods

### Recruitment and sample selection

Qualitative interviews provide the best avenue to explore people’s experiences and perceptions [34]. Exploring people’s experiences enables understanding of how people conceive health and illness generally [35] and in this study, how those in our study sample experienced workforce participation and mental health and wellbeing.

To recruit people in the paid workforce who had experienced low mental health, we used a South Australian population-representative survey. We commissioned a series of questions for inclusion in the 2009 South Australian Health Monitor survey about respondents’ paid work, including their employment arrangements, working conditions and self-reported health. The survey had 1,853 respondents and was administered using computer-assisted telephone interview (CATI). We also included a question seeking respondents’ permission to be contacted by the research team for a one-off face-to-face interview about their employment, health and wellbeing. Our respondents were selected from those responding positively to this question and were recruited according to the process detailed below.

Our study was designed to examine the experiences of people who reported low mental health and were in paid work so we selected for study inclusion respondents who, according to their survey responses, were in paid work and had a Mental Component Summary score (MCS) of less than 42 according to the SF-12v2 [36,37]. We used MCS <42 as a predictor of poor mental health because responses that score below 42 are commonly considered indicative of diagnosable mental illness [38]. The SF-12v2 used was a standard recall version (4 week) which reflects that the participant at the time of the survey and/or weeks leading up to it was experiencing low mental health. This measure was primarily used as a tool for recruitment for the current study. The interviews allowed for an examination of the source and the extent of participants’ low mental health and how their mental health might be affected by interactions between domains.

Sixty six respondents in paid employment and with MCS below 42 agreed to a further interview at the end of the telephone survey. Authors one and two recruited and interviewed study participants, having attempted to contact all survey respondents matching the selection criteria on their preferred telephone number at least three times. We did not interview 35 because we could not establish contact, eight refused to participate and two were no longer working. Nevertheless, we collected sufficient data to answer our research questions and the final few interviews indicated that new themes relating to mental health and work were not emerging. We obtained ethics approval from the Flinders University Social and Behavioural Ethics Committee prior to conducting this study. We provided verbal information about the study at initial contact and followed this up by posting a letter of introduction from a project Chief Investigator (author four), an information sheet outlining project aims and detailing what participation would entail and a consent form. Interviewers gained written consent from participants at the time of each interview. The study was conducted in accordance with the Declaration of Helsinki.

### Sample description

The sample comprised 10 females and 11 males (Table 1). Half of all participants lived in Adelaide (city of 1 million) and the other half in small towns at least one hour’s drive from any metropolitan area. Only two of the men worked part-time, whereas only four of the women worked fulltime, in a diversity of occupations and skill levels (Table 1). The majority of participants (16) lived in a marriage or de facto relationship, with seven of those also living with children. MCS scores ranged from a low of 16.55 (females) and 26.1 (males) up to 42. Only one female and three males had rated their general health in the survey below ‘good’.

**Table 1:** Participant characteristics

	
**Females**	age, self-rated health status, SF-12v2 MCS score (0-100), employment status, occupation, living arrangements, locality (rural/metropolitan)
Imogen	29, good, 35.58, full-time, manager (fast food store), alone (separated) & children, rural
Kate	36, good, 37.51, part-time, hospital ward nurse, partner & children, rural
Tanya	40, fair, 40.53, part-time, administrative assistant, partner & children, metropolitan
Leah	44, very good, 40.82, part-time, cleaner, partner & children, metropolitan
Millie	44, very good, 41.3, full-time, primary school teacher, alone (separated) & children, rural
Maria	53, good, 32.53, part-time, care worker, alone (separated) & child, rural
Jessica	55, very good, 16.55, part-time, care worker, partner only, metropolitan
Rose	55, good, 33.43, full-time, secondary school teacher, partner only, rural
Isla	60, good, 40.53, part-time, sales worker (retail), partner only, metropolitan
Lily	62, excellent, 32.9, full-time, hotel worker, partner only, metropolitan
**Males**	
Hayden	23, good, 41.62, full-time, administrative assistant, lives with parents, metropolitan
Kevin	26, very good, 36.14, full-time, engineer, alone, metropolitan
Jake,	44, good, 26.1, full-time, environmental health officer, partner & child, rural
Harry	45, very good, 33.2, full-time, funeral director, partner & children, metropolitan
Charles	47, good, 29.43, part-time, sales worker (retail), partner & children, metropolitan
Nick	49, good, 41.28, full-time, sales worker (medical), partner only, metropolitan
Ethan	51, very good, 38.46, full-time, manager, public service, partner only, metropolitan
George	53, fair, 40.38, full-time, trade worker, alone (separated) & child, metropolitan
James	59, fair, 27.78, full-time, manager (manufacturing), partner & children, rural
Henry	61, good, 32.86, full-time, trade worker, alone (separated), rural
Jacob	70, fair, 39.29, part-time, trade worker, partner only, rural

### Interview schedule design

We designed a semi-structured interview schedule so that questions would be open-ended and conversational to ensure that we captured the complexity of participants’ experiences [39].

We expected that contradictions could exist between participants’ accounts of interactions among domains and their subsequent effects on mental health. Rather than see contradictions as a problem, we sought to include negative cases to ensure complexities and contradictions were visible. Indeed, as Kvale points out it is ‘a strength of the interview conversation to capture the multitude of subjects’ views of a theme and to picture a manifold and controversial human world’ [39 p.8].

Interviews began by asking about participants’ employment history, their current job and working conditions, for example, ‘can you tell me a bit more about how you are employed – what are your hours like in a typical week?’, ‘can you tell me about the relationships you have in your job?’ and ‘what do you like and dislike about the culture in your job?’ Subsequent questions focussed on participants’ health and wellbeing, their activities and relationships in other domains of their lives, for example, ‘can you tell me some more about your health and wellbeing at the moment?’, ‘is there anything else in your life that you would like to be involved with but you aren’t because of work?’ and ‘what about in the other direction. Are there things in your life that have an impact on your job?’ Interviews averaged 90 minutes, ranged from 40 to 135 minutes and were transcribed in full using pseudonyms to maintain confidentiality.

### Analysis

We analysed interview data using framework analysis because of its usability for applied policy contexts and systematic approach [40]. Framework comprises five overlapping and iterative stages: familiarisation, identifying a thematic framework, indexing, charting, and finally mapping and interpretation. Research team members read and coded several transcripts to achieve familiarisation and develop the coding framework. We developed a thematic framework, informed by the interview transcripts and the project’s *a priori* aims and focus areas, such as employment history, current paid work, terms of employment and future preferences, social and family life, and health and wellbeing. We undertook descriptive charting to provide key information about each interview, including health status, demographic information and details including employment, social activities and family responsibilities for each participant. Descriptive charting refers to creating a table (or chart) that includes brief descriptions of the participants’ experiences, rearranging the data according to the thematic content, case by case. We entered interview transcripts into QSR International NVivo 8 for analysis, coded them according to our thematic framework, and authors one and two double-coded a quarter of all transcripts to ensure coding consistency. We addressed inconsistencies by discussing differences in coding during research meetings and amending coding practice until coding was consistent. NVivo also provided coding consistency as a tool to calculate percentage agreement and to interrogate the data where there was significant mismatch. Illustrative participant quotes are used extensively throughout the findings section from the broadest range of interviews possible to provide evidence of interpretative rigour [41].

In order to explore the research questions posed in the current paper in a way that would capture complex domain interactions, the research team further analysed the transcripts according to participants’ narratives about how their experience of interactions among life domains affected their mental health. We selected four vignettes (two males and two females) that were illustrative of the types of experiences of domain interactions reported by participants. Vignettes are defined by Miller and colleagues as ‘the researcher’s account of the relevant or core elements and recurrent themes’ [42] p.207] of a participant’s experience.

The data collection and reporting adheres to the RATS guidelines on qualitative research (http://www.biomedcentral.com/authors/rats).

## Results

Participants’ accounts indicated that the psychosocial environment associated with their paid work affected their mental health, with workplace relationships, employment security, control over number and scheduling of hours, and wages and conditions being the most salient influences. We expand on these factors below and then, using four vignettes examine how interactions between life domains of paid work, family, community, and support services explain how psychosocial work environments affect mental health. Further, we show how these impacts were related to time, space, life-stage and power.

### The psychosocial work environment and mental health

Participants described the complexity of the ways in which their environment at work affected their mental health, citing both harms and benefits. Most talked about links in terms of paid work causing ‘stress’, or expressed anger or distress while talking about particular workplace events or conditions rather than stating outright that their poor mental health resulted from their experience of paid work. Some did speak directly about ways in which their job affected their mental health, for example by explaining they were taking medication for depression in response to workplace events. Very few described paid work as being solely beneficial for mental health, or as the major contributor to their low mental health status.

### Workplace relationships

Participants who experienced bullying or discrimination in the workplace were the most likely to describe direct adverse effects of employment on mental health and to report being diagnosed with, or taking medication for depression and/or anxiety. Ethan, a public servant, described being bullied by a manager and his experience of subsequent exacerbation of poor mental health.

Last year I had three months off following some issues with a manager and that was, yeah, not a good time for me. I was three months off work then for three months I had to be placed into another area because my doctor did not allow me to work with this individual (Ethan).

While Ethan reported being diagnosed with depression about 20 years prior to the interview, he had not been taking medication ‘for many years’ but had returned to using medication after this bullying episode. He sought assistance in the work place but was redeployed rather than his manager being required to change her behaviour. He described using other coping strategies: changing his attitude regarding the importance of paid work in his life, finding solace at home with his partner and his dog, meditation and participating in physical activity:

Well there was [partner] obviously. My dog - and I got back into doing meditation and exercise as well so I think a combination of all those sorts of things helped. I think probably back then I made a decision too that well it’s only work - it’s only a portion of my life (Ethan).

Other participants reported that good working relationships were part of what they enjoyed about their job, for example George, a labourer, said ‘I might sound old fashioned but I like the camaraderie, the lads are really good for the most part’.

### Employment security

The level of employment security experienced by participants was crucial to how they managed their daily lives and consequently affected their mental health. Lily, a hotel employee working mainly as a kitchen hand, reported that other than those in management positions, most hotel employees were employed on a temporary basis. This meant their employers could reduce their hours to encourage them to leave, indicating the degree of insecurity of hotel industry jobs.

They don’t sack you nowadays they cut your hours or they make you feel uncomfortable that you want to leave […]. If you say too much in there you’re out the door, so that’s the way it is (Lily).

Lily experienced sexual harassment at work and at her daughter’s insistence had made a complaint, although she was worried she would lose her job, stating ‘I was scared because I needed a job’. Lily was paying half of her daughter’s mortgage and expressed concern that her daughter would lose her house if she lost her job, especially because she felt she had low employability: ‘I’m old, you know, I’ll not get another job’.

Like Lily, those participants who viewed their employment as insecure and their chances of finding another job as low typically described experiencing distress, particularly if they had financial responsibility for others. For these participants, losing their current employment would have significant repercussions, including being unable to meet loan repayments or having to relocate, meaning unwanted disruption to their families.

Participants who experienced job security, by comparison, described feeling good about this, with some describing mental health benefits. George, a labourer, had previously worked as a casual employee and felt that having permanency was beneficial for his mental health in spite of worrying that he could still be made redundant.

It seems to crop up every year or so ‘there could be redundancies’. [It] is really quite a good feeling to have a permanent employment for that long and long service and everything else that’s built up […]. With permanency there’s always that feeling of comfort there (George).

### Number and scheduling of hours

Having flexibility in employment arrangements, working days or working hours was seen as beneficial to mental health primarily because of family responsibilities as Jessica (a care worker) account indicated:

I was doing two nights [per week] and I felt that it was affecting my health, with depression – because I was doing a lot of other work – other babysitting for my daughters, grandchildren and things, and so I asked to drop one night and work a day instead so I’m now working two days and one night […]. So yeah that was flexible and I was pleased with that (Jessica).

Similarly, for some participants the increase in potential for flexibility resulting from technological advances has enabled them to undertake paid work at home, reducing their hours away from family:

60 hours wouldn’t be now ‘at work’. […] Every day I’m home with the kids in the morning, have breakfast, sometimes I drop them off at school […]. With a laptop, mobile, I can be anywhere and still do the job I need to do. […] It’s been fantastic (Harry).

Taking paid work home was not helpful for other participants though, particularly where they perceived their workload was too high or hours too long or where paid work permeated other domains to the extent that it affected their mental health. Nick, a sales representative, illustrated this point, stating: ‘I don’t want to be home working, I’d rather be with the family’.

### Wages and conditions

The working conditions that participants were most likely to report as having negative effects on their mental health were: dissatisfaction with pay, lack of conditions such as breaks at work, having to work while sick, and having little control over their working conditions often because of coexisting job insecurity and/or low employability.

Conditions regarding pay that participants reported negatively affected their mental health included: not being paid for overtime (or at overtime rates), not getting performance-based pay increases or promotions when warranted, and inequitable pay between staff in comparable positions. Harry, a general manager, described feeling angry because performance reviews were not considered when his company considered promotions and pay rises:

The performance reviews we have [are] not really worth anything because the company doesn’t take into account those, even for promotions which, in my view, is ridiculous. […] I just got a letter last week, as an example, my salary went up 1.8 per cent and the salary I’m getting now, even with that increase, is still less than what my predecessor got. It’s like a slap in the face. It’s ‘hang on, you’re telling me I’m not even worth what he was two years ago’ (Harry).

Like Harry, participants’ accounts typically revealed disappointment in terms of feeling they were not valued by others – either by their employer or by society. Participants in low-skilled jobs reported that being unable to take breaks or sick leave affected their physical and mental health. Isla, who worked cash-in-hand, without an employment contract, as a shop assistant, described not being able to take any breaks over the course of a workday:

[If I asked for a break he would say] ‘if you’re not going to work under my conditions well you have to go’. He wouldn’t accept that I needed a half an hour break, no […] Sometimes I get a bit upset – sometimes I get all ready to go to the toilet and I’ve locked up everything and made it secure […] and I’m just about ready to go and someone walks in the shop and I’m like ‘oh I can’t go’ (Isla).

These participants described feeling that their roles were not valued, Isla stating: ‘shop assistants, I really think they’re not treated very well […]’.

Participants who reported having access to leave and/or breaks as standard, by comparison, tended to be those who were more educated and/or whose skills were in high demand, for example, teachers and nurses.

### Interaction between domains & mental health

The psychosocial environment people experienced in paid work affected their experience in other life domains and their other life domains either ameliorated and/or exacerbated their poor mental health. Participants described four main life domains affecting their mental health: paid work, family, community and support services, as well as the broader socioeconomic and political macrosystem. While Voydanoff’s socio-ecological model [8] collapses services (e.g. private and public health and education services) in to the community system, our findings suggest that while support services may be located in the geographical community, they were separate from the community in the sense that they were not part of everyday social relations and usually involved payment.

We conceptualised the four domains in terms of participants’ relationships within them, the spaces where particular activities took place, and time spent on activities specific to particular domains. Participants’ accounts typically illustrated domain boundaries as fluid, permeable or malleable. The paid work domain, for example, included all spaces where participants described undertaking work activities (including thinking about work) and thus was not restricted to the physical workplace. The home domain included the material home and also family relationships with those cohabiting and living elsewhere, such as adult children and participants’ parents. The community domain encompassed public spaces where participants undertook civic participation such as voluntary work and daily tasks of shopping for household groceries, and social networks including talking with neighbours or participating in recreational activities outside the home. The service domain comprised participants’ engagement with fee-for-service and state-funded public services such as private health professionals, state-funded health services, childcare centres and schools.

The ways in which domains interacted and the subsequent effects of these interactions changed over time, as part of the participants’ life stages and their experience gained over time regarding how best to manage their mental health. The complexity of the domain interactions are best illustrated through more detailed accounts of our participants. Using four vignettes we illustrate how participants described managing their mental health in the context of their multiple life domains. We then further unpack the influences of time, space, life-stage and power [30].

### Four vignettes: experiences of domain interactions

#### Kate

Kate, a nurse employed via a temporary agency, was the participant who described paid work as being most beneficial for her mental health. Kate’s experience of interconnections between paid work, family, community and services illustrates the ways in which mental health is affected by all four domains in interaction. Kate had experienced postnatal depression after the births of her two children, the first being born while she was living away from her family, a community and services she was familiar with and the second when she was living close to these. Consequently, her two postnatal depression experiences were very different but paid work also played a key role in ameliorating her mental health problems.

Kate’s first experience of postnatal depression was exacerbated by a number of factors, including: isolation resulting from a move away from her family and social networks for her husband’s job prior to their baby’s birth, not being in paid work herself because of having a new baby and not knowing what was wrong with her mental health until a child and youth health nurse diagnosed postnatal depression:

I had a first mum’s group […] and it was the nurse at that that picked up something was amiss with me. […] and then I was hospitalised with [baby] and she worked at the hospital […]. That was just huge, to have someone go ‘look, it’s okay, you’re normal’.

Prior to the birth of her second child, Kate’s family moved nearer to her parents and long-standing social connections:

Just the isolation and if we were to have another baby and I was isolated again I would have – my world would have come crashing down […]. It’s a dark, scary road; very dark, scary road. But it’s all good now.

Kate comment that she ‘wasn’t as bad the second time around as I was the first time’ refers to experiencing less severe post natal depression the second time and she attributed this to having access to support from family and services:

Knowing I had support. I knew where to go; [interstate] I didn’t know where to go. Here I’d sought out where I go [interstate] we didn’t really have a GP, so here I had a GP. So it was all that stuff that you take for granted I suppose. […] If I didn’t have my health nurses that I had with both the girls who knows how sick I could have got.

Further, Kate found that returning to paid work as a nurse soon after her second baby was born benefited her mental health. She had initially intended to work for only two months for one day per week, but decided to continue working and later increased her hours.

I think going back to work so soon really helped, otherwise I would have been stuck at home with a four and a half year old and a 12 week old so going back to work and getting that one day a week break was fantastic for me […] I got out of the house, it gave me a reason to get up in the morning; I was out there and I could be myself, I didn’t have to pretend to be this fantastic mother, I could just be this nurse that I knew how to be. At that stage, during that time, it was very good therapy.

Kate found little conflict between domains because she could specify her working hours so they did not impinge upon her family responsibilities and because her mother helped with childcare:

Nursing is just so […] flexible and fits in with family life […]. It just works well for me, for us. We don’t need to stress about out of school care, we don’t have to stress about stuff like that, so that’s good for our health too because we’re not stressing about all those extra expenses […]. But mum gets them on the two days – well three days a week now mum just picks them up and they do their homework and then I’m there.

Thus, Kate’s experiences of postnatal depression were influenced by the interconnections of four domains: 1) family (gendered responsibility for children), 2) support services (child and youth health nurse, family GP), 3) community (broader family and social networks) and 4) paid work (a break from mothering). Time and experience were also important factors: Kate learnt from her first experience of depression and took preventive action before her next child was born. She was able to do this because of her high employability and flexibility as a registered agency nurse, previously established social networks and the dependable childcare her mother provided.

#### Rose

Rose, a school teacher, gave an account of the relationship between mental health and life domains that was typical of several of the women who juggled family, paid work and community responsibilities and also of participants whose paid work permeated other life domains in a way that was unwanted, in spite of gaining a great deal of satisfaction in the work itself.

When Rose’s children were young (at interview they were adults), she was living in a country town, driving her two pre-school aged children to childcare in another town and then driving to the school where she was teaching. The state education system is centrally controlled and neither schools nor teachers have much influence regarding school placements, staffing levels or workload. The interactions between Rose’s family (gendered role caring for young children plus children’s responses), community and her paid work culminated in Rose experiencing a high level of stress to the point that she felt she had to make changes to protect her health or resign.

I had two young children at that time, very young, and I just wasn’t handling the fact that I was living in one town and having to drop my kids off in another town and then go to another school and work. I was part-time but I was needed there every day and I was just suffering a lot of stress from it all and I thought no, I can’t handle this anymore, and my son was getting into trouble at kindergarten because he was being a rat-bag […] it was just awful.

She sought the support of a union representative who arranged for her to be placed in a school nearer to her home. This input from the union representative damaged her relationships with her previous school principal and colleagues, but the change improved her mental health.

When interviewed Rose reported conflicts between domains following her move as she felt that teaching and living in the same community made her feel more accountable to her students and their parents and consequently she worked long hours to ensure she was doing the best job possible.

I’m a person who likes to do things properly and living in a country community […] I try and help other kids because they are someone’s child and some mum will appreciate it and you feel very accountable to the local community. In fact it’s very common that you have a parent interview in Woolworth’s, so wherever you go. It might be one of your ex-students wants to chat to you when they’re on the checkout. It could be anything and you do feel very connected and that’s a very important part, a positive of your job, but I think it makes you think ‘right I’m not finished yet, I’ve got to do that’

Rose described feeling personal satisfaction when she felt she was meeting her own expectation to be a high quality teacher, but this came at a cost regarding interactions between her paid work and family domains and subsequently her health. She described her own and her family’s worry about how her overworking and high stress were affecting her physical and mental health. She described bringing paid work home so that she could complete it after attending to her family and taking care of her physical health, which meant that ‘midnights are common’. When she did take time out, for example walking the dog, she felt the combination of paid and family workload weighing upon her exacerbated her stress when she returned home. She reported having high blood pressure (which was medicated), difficulty sleeping, experiencing high levels of stress, had taken anti-depressants for 14 years and had recently spoken with her doctor about starting medication to help her sleep:

Some days […] I get home and I’m thinking ‘damn I’ve got so much work to do’ and now I’m quite stressed by the time I get in the door and grumpy that I actually went for a walk and I let myself take a whole hour and now I’ve got to cook tea or get some more school work done, so it varies. […] It’s just annoyance at circumstances that I just have work to do and I took too long on my walk. […] I have trouble switching off until I think I’ve done something the best that I can and if I make what I consider a mistake or whatever I suffer huge anxiety and stress over it because I guess I’m a bit of a perfectionist […].

At the time of the interview, Rose had reduced her hours from full-time to four days a week recognising she was in the financial position and at a stage of life when she was able to reprioritise her domains:

I’ve got a couple of grandchildren due any day, any week, and my family’s much more important to me than a job. I guess I’m in a fortunate position where financially I don’t have to bring in a big income. I think it gives me much more freedom to say ‘I do this because I want to, I’m lucky’.

In summary, Rose experienced multiple domain interactions which had affected her mental health at different life stages. At the time of her interview, paid work infiltrated all of her domains: 1) family (family worry about work impacting on her health, competing demands at home, work interrupting sleep), 2) community (meeting with students and teachers while shopping) and 3) support services (seeking help from her doctor about paid work stress causing insomnia). Nevertheless, Rose experienced personal reward when she felt she was able to achieve the high standards she set for herself and was able to reduce her work hours because her family was in a strong financial position.

#### Nick

Nick’s account of working as a sales representative was typical of those participants who had few resources to draw on in order to make changes in the paid work domain to support their mental health and participation in other domains. He described conflict in interactions between paid work and family and also the limits that his paid work placed on his community participation. He reported employment insecurity and low employability that he associated with his age (nearing 50) and gender, rather than skill or performance, stating that: ‘I’m anticipating that I could be unemployed within anywhere from four weeks to four months’ and that: ‘basically, for want of a better word, [if I lose this job] there is nothing’. He described macrosystem changes in the industry he worked in, whereby large corporations were ‘swallowing up’ smaller and previously locally owned companies and organisational restructuring:

We went through another restructure and they actually announced three months before that they were going to cut 25 per cent of the staff, so that sent everybody through the roof, particularly me. I think I was really affected by it and I was on holidays […] the worst time I’ve ever had on holidays. I think I probably had maybe 15 to 20 hours sleep for a whole week […]. And the situation was so bad I sought medical intervention with my doctor(s) and my boss thought that was – she thought that’s a weakness […] even though I was still showing up to work and still doing the work.

Nick indicated that he had been so stressed about his relationship with his new manager that he had thought of suicide:

I’m treated more harshly in meetings or anywhere else by the manager […] the stress got so much a little while ago that – I was probably 10 seconds from doing something very out of the ordinary and so I just stopped the car and I rang my doctor [and he] made time for me to go and see him straightaway.

Nevertheless, he also felt that being able to talk to colleagues was of some help, stating that: ‘the rest of my team really like me […] they feel exactly the same as I do with the stressful situations’. Nick attributed his poor mental health to a combination of workplace relationships, poor job security, and the repercussions that would follow for his family if he lost his job:

[My son and daughter-in-law] bought the house and he started getting cutbacks and all that type of thing [my daughter] works as a hairdresser so she’s tried to get extra hours and all that sort of thing but that’s all very difficult to do because everyone’s pushing for the extra hours […]. Her kids are younger so therefore they need childcare or family so yeah I’ve had to take a second mortgage out so that they can keep their house.

He had been involved in community activities such as team sport and also coaching sports and had played cricket until the year before the interview, but had stopped due to workload. Nick reported having the option to work from home, or to take on other paid work that involved more travel, however he did not want to move away from family and wished to keep his home life separate from paid work:

You can actually apply to work from home part of the time or a percentage of the time which – if you’re in sales you can’t make a sale unless you’re actually seeing the customer so that’s pretty difficult to do and as it is when you’re paid to work 40 hours a week and you’re working 52 to 58 hours then I can’t see the real change.

To summarise, Nick’s account indicates that his mental health was adversely affected by a poor psychosocial environment associated with his job: he was being bullied, felt undervalued and experienced high job insecurity. His account suggests that his poor mental health was exacerbated and ameliorated by domain interactions. These included: 1) consequences for his family if he lost his job, particularly his adult children who relied on his income to assist with mortgage repayments, 2) limited time to participate in the community domain (sporting activities) because of high workload, and 3) accessing help from his doctor to manage his mental health. Unlike Kate and Rose, Nick described having little power to change his circumstances without further exacerbating domain conflict, for example by increasing paid work-related travel or working from home and had few alternative financial resources.

#### Kevin

Kevin, an engineer, provided an account illustrative of the experiences of the few participants who were single and/or without dependent children. Kevin’s experience of domain interactions mainly involved the ways in which paid work impinged on his social life and how the stress of his job stayed with him while not at work. He had suffered depression following his parents’ separation and stated: ‘it’s always at the back of my mind that it could come back so now I’ve taught myself to recognise the thought pattern which triggers it so I try and change that’. He felt that while paid work did not directly influence depression, when he ‘felt down’ his work performance suffered which consequently exacerbated depression:

It does definitely, like my productivity goes down quite a lot and […] if I’m feeling down I’m not working as well which in turn makes me feel even more down because I feel I should be producing a better output of work.

When he started in his current employment he had a supervisor who could train him. Six months in, however, his supervisor left and so he was required to fulfil both roles and as the only employee qualified in his field felt that: ‘the company’s success rides on my shoulders to a fair extent’. He reported that initially, he did his best to keep up with the high workload stating: ‘I’m a perfectionist; it’s a shit trait but that’s where I am’ and experienced stress about his workload, which permeated into his other domains. He felt that high levels of stress were potentially detrimental to his health, so like Ethan’s account in the previous section, over time he changed the way he thought about paid work:

Oh it caused me a lot of stress for a long time. I’d come home from work and I’d just – I was tearing my hair out ‘I’ve got so much work to do, so much work to do’. Now I’ve still got that much work to do, if not more, but I’ve just stopped caring. It’s not worth worrying about you know, you’ll give yourself – oh God only knows what. It can’t be good for your health stressing all the time so I just stopped stressing.

He elaborated that he also felt less obligation to his employer because he had not had his annual performance review and subsequent pay rise. In addition, because he received a flat salary, the overtime and Saturday work he did was not remunerated:

When I first got promoted the boss was like ‘work every second Saturday’ […]. I guess I feel like why should I have to work Saturdays if I don’t get paid any more money for it? Everyone else there is getting paid overtime for it, I’m the only one that doesn’t because I’m in a different company, so it’s like what’s the fucking point?

Kevin described enjoying recreational activities such as team sport, water sports and spending time with friends and expressed resentment that working overtime, particularly working on Saturdays, reduced the time he could spend participating in these.

Kevin’s account contrasts with the previous three because he was single and did not have direct caring responsibilities or other family demands. The main domain interactions he described harming his mental health were 1) paid work-community interactions (being prevented from participating in recreational activities) and 2) high workload mixed with feeling he was not valued by his employer (reflected in the lack of overtime pay for weekend work) affecting his mental health while at home. He had taken action to reduce these interactions by resisting working on Saturdays and by decreasing the importance he had previously accorded to his paid work. As a university graduate in an industry where people with his qualifications were in demand, Kevin had the power to be able to be proactive in limiting conflict between domains.

### Time, space, life-stage, power and mental health

Our findings indicate that time, space, life-stage and power cut across the four domains of paid work, family, community and access to support services [30] and interacted with the psychosocial environment associated with paid work to influence mental health. Further, as we describe below we found these themes to be intertwined; particularly the first three – time, space and life-stage.

### Time, space and life-stage

The extent to which time spent in each of the domains fitted with the participants’ lives, including their (often gendered) demands and supports, such as caring responsibilities and partner’s paid work, affected participants’ mental health. Kate’s account demonstrates how time at paid work can be beneficial because it reduced her time in the physical and emotional space of the family domain, ameliorating the effects of postnatal depression. Rose’s and Nick’s experiences, by contrast, demonstrate how long working hours can affect both physical and mental health; for example both found that time for participation in physical activity was reduced, or in Rose’s case became an added stress if she did spend time (that she didn’t have) walking. Kevin also found that paid work impacted adversely on the time he had available to participate in recreational activities.

Some participants found that the creation and maintenance of separate spaces for domains could be beneficial for mental health, for example the travel time between spaces (particularly those of workplace and home) being a preparation time on the way to work and a wind-down time on the way from work to home. For others, the encroachment of one domain space into another (paid work being undertaken at home for example) was positive for mental health in allowing them more flexibility in moving between domains (for example, being home with children at meal times).

Life-stage was very important to how participants managed interconnections between domains. Rose’s experience of having to travel between spaces (home, childcare and paid work) within a limited time-frame while her children were young reflected her life stage as a mother with young children. Participants emphasised the importance of learning from previous experience to manage their mental health as well as how domain interconnections affected their mental health. Kate’s learning from her first experience of postnatal depression meant that she was better prepared for her second experience.

### Power

The level of and access to power and control participants had at work shaped their experiences of mental health across domains. Power in the paid work domain underpinned the job qualities that impacted on mental health the most: workplace relationships, employment security, control over number and scheduling of hours and wages and conditions. Power could be exerted to change or control the psychosocial environment in the paid work place when people were in a better bargaining position and/or had access to avenues to facilitate change (e.g. workplace policy or union representation).

Nick and Isla felt that their insecure employment meant that complaining would result in losing their job and participants believing this did not attempt change. Ethan, by contrast, as a management-level public servant knew he could use formal complaints mechanisms to address his experience of bullying by his manager without losing his job. Nevertheless, his complaint resulted in him being redeployed rather than his manager being required to change her behaviour.

A few participants who felt they had little power to improve paid working conditions or relations drew upon the assistance of workers’ unions, with mixed outcomes. Lily reported drawing on union support on two occasions. Firstly she successfully sought employer superannuation contributions and back-pay, for which she was eligible but had not been paid. Secondly, she attempted to oblige her employer to take her sexual harassment complaint seriously, as complaining directly to management had not worked:

I could have done it myself, but as I say to do it yourself, they don’t listen to you and fob you off, but if you’ve got a union person there, they have to listen and they have to do the right thing (Lily).

The only way she could resolve the sexual harassment, however, was to leave her employment and so she sought work in another hotel run by the same company because no action was taken against the perpetrator.

Isla had sought union assistance in her previous job after she was told not to return to work (which was a temporary position in a large retail store) after breaking her leg and having time off. While the union won her job back, she was repeatedly refused permanency and experienced bullying from her manager because she was labelled a ‘troublemaker’. George’s use of union representation, by comparison, helped him to secure a meaningful position after workplace injury, without adverse repercussions.

## Discussion

This study enabled exploration how individuals with low mental health navigate the workplace and other domains, and the strategies they use to protect their mental health. Our findings suggest that the psychosocial environment associated with paid work can strongly influence mental health positively and/or negatively. Examining this environment alone, however, does not provide a full picture of how people with low mental health are able to manage their health and balance competing life demands. In our sample, the mental health effects of paid work were shaped by interactions with other life domains notably family, community [8] and access to support services while Pocock’s [30] cross-cutting themes of *time*, *space*, *life-stage* and *power* added further complexity to these interactions. We therefore answer our research questions regarding mental health effects of the psychosocial work environment and domain interactions together.

### Mental health in multiple domains

Workplace relationships were reported as the most important workplace influence on mental health, supporting evidence that interpersonal conflict in paid work is the major workplace stressor across occupations [12]. In Australia, workplace legislation requires organisations to provide formal complaint mechanisms to deal with discrimination, bullying and sexual harassment [43]. In spite of this, participants reported that their workplaces failed to address interpersonal conflict such as bullying, sexual harassment and discrimination (even when there were formal complaint mechanisms and they had used these) and that this failure had detrimental effects on mental health. Some responded to the failure of employers to address these issues by drawing support from other domains, including seeking medical help, and/or by withdrawing some of their effort from paid work and putting more energy into community and/or family activities. Our findings suggest that promoting positive workplace relationships and strengthening workplace complaints mechanisms to provide greater support to complainants could improve workers’ mental health.

We found that insecure jobs affect workers’ mental health because of worry about the repercussions of losing their job and the subsequent loss of income. Mental health effects of insecure employment were exacerbated for those who had family financial responsibilities and anticipated consequences such as adult children being unable to meet mortgage repayments or having to move house or children having to change schools. Mental health effects of job insecurity were particularly strong for those participants who perceived it was unlikely they would find another job, supporting evidence that job security and employability are interrelated with mental health [2,16,18,20,44,45] and indicating that these effects should be considered in the context of domain interactions. There is evidence that in nations that provide higher levels of social protection (e.g. Scandinavian countries), workers experience lower levels of workplace psychosocial stress than those living in nations with weaker social protection systems (e.g. southern Europe) [10]. Strengthening the Australian welfare system (rather than the current policy push to reduce welfare expenditure), particularly in the face of employment insecurity, could therefore ameliorate poor mental health experienced by those in paid work and the unemployed.

Our study clearly reveals how mental health is affected by complex interactions between domains and that these interactions may be ameliorated or exacerbated by time, space, life-stage and power. We add to evidence that having control over the scheduling of hours spent in paid work is important for maintaining positive mental health, and potentially more important than the number of hours worked, especially for those workers with family responsibilities [45]. Gender-based differences in the effects of interactions among domains were not especially evident in this study. It is worth recognising that in this respect our findings differ from those of some other studies. Recent Australian research shows that women carry the larger burden of unpaid domestic labour, especially child care [46]. Women are also more likely to be engaged in lower paid, lower skilled occupations and more likely to be in part-time or casual employment [7].

Time is important because scheduling of hours spent in paid work influences the number of hours and time/s of day that are available for activities in other domains. Where time, space and life-stage intersect (for example parents taking paid work home) to enable people to spend more time with their children, there are potential mental health benefits. Conversely, if taking paid work home means unwelcome intrusion into other domains, it is perceived as detrimental to mental health. This includes worry or stress about work while at home, such as being unable to stop thinking about paid work while at home and leading to effects such as insomnia. Thus, having the power to negotiate the extent to which paid work is done outside formal working hours is good for mental health. Finally, we found that wages and conditions were interconnected with other aspects of the psychosocial work environment and their interaction with other life domains affected workers’ mental health. Those participants who felt their jobs were insecure did not seek improvements to their wages or conditions for fear of losing their jobs. Thus, life-stage and lack of bargaining power were potent influences on the mental health of participants in insecure jobs with poor wages and conditions. Membership of trade unions was important for negotiating conditions, particularly those participants with little power within their workplaces but not all workers have access to union support.

People in low skilled, low paid jobs with job insecurity and poor working conditions also had the least power to change the psychosocial environments associated with paid work in order to improve their mental health. On the other hand, some participants (e.g. Rose) illustrated that having benefits such as lunch breaks did not always translate into being able to use them. Elsewhere, we have found that those workers in skilled occupations and working through temporary agencies may, in some circumstances, experience better mental health than their permanently or otherwise more securely employed colleagues [47].

### Strategies to maintain mental health: Implications for workforce and social policy

Our study suggests that workers use multiple strategies to protect their mentals health at work. A small number of participants used formal mechanisms such as workplace policies or unions, to initiate changes to improve conditions. In other cases although these formal structures were available, people’s attempts to use them to improve their work environment were unsuccessful. Others either did not have the power to use these structures or they were not available. Participants also used informal strategies to manage their mental health. Some participants changed their priorities, for example by reducing their psychological commitment to paid work. Some who reported doing this also felt reduced commitment and loyalty to their employers, that is, they experienced a break in the psychological contract between employer and employee [48,49]. Like other studies [12] we found that workers withdraw into activities other than paid work as a coping mechanism in response to workplace stressors to protect their mental health. Participants who were able to instigate changes at work were typically in higher skilled, better paid jobs than those who were not, once again demonstrating that the effects of the psychosocial environment of paid work on mental health reflects the power workers have available to them.

We acknowledge that our categorisation of individuals as having low mental health used a relatively crude instrument (i.e. the SF-12v2 is not a clinical assessment tool) and that mental health is fluid, with an assessment at one point in time (the survey) not necessarily applying to another time period (the interview and beyond). Further, we interviewed those survey respondents who we were able to contact and were willing to participate, meaning that the range of participants may have been limited. However, the interview material did indicate current mental health issues for participants and provided considerable insight into how these issues were affected by, and managed, in paid work and in the rest of life.

## Conclusions

Our findings suggest that there is considerable benefit in considering the mental health effects of the psychosocial work environments experienced by people with low mental health in paid employment, alongside interactions within and among other life domains and in terms of time, space, life-stage and power [8,30]. Workplace policy has a central role to play in promoting workers’ mental health. Individual strategies to cope with the mental health effects of poor psychosocial environments in the workplace were helpful for participants, but strengthening workplace policies to promote good mental health may have other benefits such as improved productivity. Promoting positive workplace relationships and in particular, providing effective mechanisms to deal with bullying, discrimination and sexual harassment, and protective mechanisms that enable workers to make changes or complaints without detrimental repercussions (such as vilification or job loss) would go some way toward improving mental health in the workplace and consequently across other life domains.

## Competing interests

The authors declare that they have no competing interests.

## Authors’ contributions

CRM and DK conducted interviews, analysed data, contributed to planning the structure of the article and drafted the manuscript. AMZ, FEB and JP conceived of the study, analysed data, contributed to planning the structure of the article and provided critical revision of the manuscript. All authors read and approved the final manuscript.

## Pre-publication history

The pre-publication history for this paper can be accessed here:

http://www.biomedcentral.com/1471-2458/13/796/prepub
